# Strengthening Global Health Security Through Africa's First Absolute Post-Master's Fellowship Program in Field Epidemiology in Uganda

**DOI:** 10.1089/hs.2018.0045

**Published:** 2018-11-27

**Authors:** Alex R. Ario, Rhoda K. Wanyenze, Alex Opio, Patrick Tusiime, Daniel Kadobera, Benon Kwesiga, Lilian Bulage, Christine Kihembo, Steven N. Kabwama, Joseph K. B. Matovu, Steve Becknell, Bao-Ping Zhu

**Affiliations:** Alex R. Ario, PhD, is the Field Coordinator, the Uganda Public Health Fellowship Program, Ministry of Health, Kampala, Uganda.; Daniel Kadobera, MSc, is Field Supervisors, the Uganda Public Health Fellowship Program, Ministry of Health, Kampala, Uganda.; Benon Kwesiga, MPH, is Field Supervisors, the Uganda Public Health Fellowship Program, Ministry of Health, Kampala, Uganda.; Lilian Bulage, MHSR, is a Scientific Writer, the Uganda Public Health Fellowship Program, Ministry of Health, Kampala, Uganda.; Rhoda K. Wanyenze, PhD, is Dean and Program Director, Uganda Public Health Fellowship Program, Makerere University School of Public Health, Kampala, Uganda.; Steven N. Kabwama, MSc, is Training Manager, Uganda Public Health Fellowship Program, Makerere University School of Public Health, Kampala, Uganda.; Alex Opio, PhD, is Commissioner Emeritus, National Disease Control, Ministry of Health, Kampala, Uganda.; Patrick Tusiime, MPH, is Commissioner, National Disease Control, Ministry of Health, Kampala, Uganda.; Christine Kihembo, MIPH, is a Scientific Writer, African Field Epidemiology Network, Kampala, Uganda.; Joseph K. B. Matovu, PhD, is a Lecturer, Makerere University School of Public Health, Kampala, Uganda.; Steve Becknell, MPH, is Deputy Country Director, US Centers for Disease Control and Prevention, Addis Ababa, Ethiopia.; Bao-Ping Zhu, MD, is Resident Advisor, Uganda Public Health Fellowship Program, US Centers for Disease Control and Prevention, Kampala, Uganda.

**Keywords:** Post-master's degree, Field epidemiology training, Global health security

## Abstract

Uganda is prone to epidemics of deadly infectious diseases and other public health emergencies. Though significant progress has been made in response to emergencies during the past 2 decades, system weaknesses still exist, including lack of a robust workforce with competencies to identify, investigate, and control disease outbreaks at the source. These deficiencies hamper global health security broadly. To address need for a highly competent workforce to combat infectious diseases, the Uganda Ministry of Health established the Public Health Fellowship Program (PHFP), the advanced-level Field Epidemiology Training Program (FETP), closely modeled after the CDC's Epidemic Intelligence Service (EIS) program. The 2-year, full-time, non–degree granting program is the first absolute post-master's FETP in Africa for mid-career public health professionals. Fellows gain competencies in 7 main domains, which are demonstrated by deliverables, while learning through service delivery 80% of the time in the ministry of health. During 2015-2017, PHFP enrolled 3 cohorts of 31 fellows. By January 2018, PHFP had graduated 2 cohorts (2015 and 2016) of 19 fellows. Fellows were placed in 17 priority areas of the ministry of health. They completed 153 projects (including 60 outbreak investigations, 12 refugee assessments, 40 surveillance projects, and 31 applied epidemiologic studies), of which 49 involved potential bioterrorism agents or epidemic-prone diseases. They made 132 conference presentations, prepared 40 manuscripts for peer-reviewed publication (17 published as of December 2017), and produced 3 case studies. Many of these projects have resulted in public health interventions that led to improvements in disease control and surveillance systems. The program has produced 19 issues of ministry of health bulletins. One year after graduation, graduates have been placed in key public health decision-making positions. Within 3 years, PHFP has strengthened global health security through improvement in public health emergency response; identification, investigation and control of outbreaks at their sources; and documentation and dissemination of findings to inform decision making by relevant stakeholders.

Sub-Saharan African countries have historically faced chronic weaknesses in the public health system, such as poor infrastructure, limited diagnostic capacity, insufficient information systems, and shortage of skilled human resources for public health (including field epidemiologists and laboratory personnel) to manage outbreaks. Production of competent public health workers has not kept pace with need, in part because of the ever-increasing burden brought about by the HIV/AIDS pandemic, the emergence and reemergence of new microbes, the upsurge in antimicrobial resistance, and the rise in zoonotic diseases due to increasingly frequent human-wildlife interactions.^1^ The spread of Ebola in West Africa during 2013 to 2016 was worsened by weak health systems characterized by lack of public health capacity for outbreak detection and control. Inadequate preparedness for response to communicable diseases outbreaks further compounded the problem.^2,3^ Empirical evidence has shown that large epidemics of viral hemorrhagic fevers such as Ebola could be prevented if countries could rapidly respond to such outbreaks, interrupt transmission, and prevent the outbreaks from spiraling out of control.^4^

Learning from past epidemics such as the Ebola epidemic, nations around the world jointly launched the Global Health Security Agenda (GHSA) in February 2014 “to build countries' capacity to help create a world safe and secure from infectious disease threats and elevate global health security as a national and global priority.”^5^ Workforce development is one of the action packages of GHSA, which recommends that at least 1 trained field epidemiologist is needed for every 200,000 population.^5^ For a population of 41 million, this translates to a need for 205 trained field epidemiologists in Uganda.

Makerere University started a master of public health (MPH) program in 1994. Over the years, it has trained 173 MPH graduates with a focus on field epidemiology. However, many of the graduates have been employed by the private sector, nongovernmental organizations, international agencies, or academic institutions. The few that are currently active in the public sector cannot satisfy the critical needs in Uganda, which has had an unusual share of outbreaks of diseases involving high-impact pathogens, such as Ebola (5 outbreaks since 2000), Marburg (3 outbreaks since 2012), yellow fever (2 outbreaks since 2010), anthrax, Crimean-Congo hemorrhagic fever, Rift Valley fever, meningococcal disease, and plague.^6-17^ Moreover, the life expectancy and healthy life expectancy of the population in Uganda rank among the lowest in the world.^18^ The country has a high burden of endemic diseases and a growing burden of noncommunicable diseases and injury. Existing human resource capacities at national and subnational levels are far from being adequate to face these challenges.

Because of the glaring gap of field epidemiologists in the public sector, in 2015 the Uganda ministry of health, with support from key partners, including the Makerere University School of Public Health (MakSPH) and the US Centers for Disease Control and Prevention (CDC), established the Uganda Public Health Fellowship Program (PHFP), the first post-master's field epidemiology training program (FETP) in Africa. It is intended to equip the country to face the aforementioned multitude of public health challenges by training and absorbing epidemiologists in service. This article describes the PHFP, its development and organization, and its contributions both to strengthening global health security at the country level and to the management of other public health challenges during its first 3 years.

## Methods

### Program Description

The PHFP is a 2-year, non–degree-granting, full-time, competency-based fellowship program modeled after the US Epidemic Intelligence Service (EIS) program. The program is designed to train midcareer professionals in health-related disciplines who aspire to become public health leaders. To qualify, candidates must have a master's degree in a health-related discipline such as public health, veterinary public health, epidemiology, nutrition, or other similar field. During the 2-year fellowship period, PHFP fellows are required to attain 6 competency domains. They attain the competencies by completing a portfolio of projects in each of these domains (Table 1).

**Table 1.** Competencies and Deliverables Required of Each Fellow of the Uganda Public Health Fellowship Program During the 2-Year Fellowship

*Competency Domain*	*Examples of Projects*	*Minimum No.*	*Deliverables*
Response to a public health emergency	Outbreak/disease cluster investigation;	4 (leading 2, participating in 2)	Reports submitted
	rapid health assessment of displaced populations		
Applied epidemiologic study	HIV/AIDS epidemiology project (required for all fellows);	2	Reports submitted
	malaria epidemiology project (required for malaria-track fellows);		
	vaccination coverage survey;		
	surveys on tobacco or alcohol use;		
	risk factors for road traffic injury		
Public health surveillance	Surveillance data analysis;	1	Report submitted
	surveillance system evaluation		
Communication	Article for Ministry of Health *Epidemiology Bulletin*	2	Articles published
	Preparing policy/media brief	1	Brief submitted
	Presenting at scientific conference	4	Presentations delivered
	Peer-reviewed publication	1	Manuscript submitted
	Writing a newspaper article	1	Article submitted
Public health programming	Economic analysis;	1	Report submitted
	quality improvement project		
Management and leadership	Leading a public health initiative	1	Initiative started and completed

A steering committee composed of key internal and external stakeholders provides oversight for the PHFP. The program is directed by representatives of 3 core stakeholders: the ministry of health, MakSPH, and CDC. The day-to-day operations are run by the program secretariat, which consists of a field coordinator, 2 field supervisors, a scientific writer, a training manager, and an administrative assistant. The CDC resident advisor, who graduated from the EIS program, has extensive experience establishing FETPs elsewhere, and has broad knowledge in medicine, field epidemiology, and biostatistics, works full-time with the secretariat. The resident advisor provided guidance on the establishment of PHFP, helped to design the curricula, participated in teaching the didactic courses, mentored the fellows directly during complex and high-profile investigations, consulted on appropriate epidemiologic and statistical methods, ensured compliance on mandatory requirements (such as human-subject protection and clearance of communication products), and served as the project officer to oversee the operations of the cooperative agreement that funds the project.

The program is primarily funded by US government through the President's Emergency Plan for AIDS Relief (PEPFAR), the President's Malaria Initiative (PMI), and the GHSA. The PHFP is an arm of the ministry of health that works closely with the Epidemiology and Surveillance Division (ESD), the Public Health Emergency Operations Centre (PHEOC), and other important public health programs in the ministry of health.

### Recruitment of Fellows

Enrollment of fellows begins with solicitation of applications through media, the program website, the alumni association, and professional associations. Short-listed candidates undergo a rigorous interview process (which includes a question-and-answer session, an oral presentation, and an essay-writing session) before being accepted. Approximately 10 fellows are selected annually. To ensure full commitment to the demanding 2 years of learning and service activities, all fellows are required to resign from their previous positions. To compensate for their loss of income, fellows are provided a monthly stipend.

### Training Approach

The learning scheme consists of 2 6-week-long didactic courses: an introductory course at the beginning and an advanced course toward the end of the first year. The introductory course covers applied field epidemiology and statistical methods, quality improvement science, cost analysis of outbreaks, effective communication, public health programming (ie, translation of evidence to public health practice), and management and leadership. The advanced course covers in-depth methods in epidemiology and statistical analysis, public health in complex emergencies, and burden of disease determination. The didactic courses are taught by MakSPH academicians, ministry of health technical staff, World Health Organization (WHO) Uganda staff, PHFP secretariat members, PHFP graduates after the first-cohort fellows have graduated, the CDC resident advisor, and other CDC technical staff at the country office. To orient mentors on mentorship guidelines and skills, the program also convenes a 1-day mentorship workshop, attended by academic and host institution mentors as well as fellows, immediately after placement of fellows at the host institutions.

For the remainder of the 2 years, fellows are placed in host sites to undergo field project–based, hands-on training. The host sites are determined based on priorities of the ministry of health, availability of learning opportunities in applied epidemiology, the fellow's desired career path and interests, availability of qualified and interested host site mentors and supervisors, and accessibility of public health surveillance data. Throughout the 2-year assignment, fellows work on priority projects, under the close mentorship of experienced public health professionals (Table 2).

**Table 2.** Roles and Responsibilities of Various Mentors of the Uganda Public Health Fellowship Program during the Fellow's Field Assignments

*Mentor Type*	*Who They Are*	*Roles and Responsibilities*
Host-site mentor	Staff members at host institution;	• Guides fellow to develop terms of reference, taking into account institutional expectations and required deliverables.
		• Provides an enabling environment for fellow to initiate, develop, and implement field projects.
	usually 1 primary and 1-2 secondary per fellow	• Guides, supports, encourages, and supervises fellow during field assignments.
		• Ensures that fellow is exposed to management and leadership experiences, challenges at host institution, and career opportunities.
		• Helps fellow to fully integrate into the host institution.
Academic mentor	Faculty members of Makerere University College of Health Sciences	• Guides and supports fellow on technical issues for the fellow's projects (eg, appropriate epidemiologic design and statistical methods).
		• Guides fellow in report writing, preparation of scientific communication products, and preparation of project proposals.
		• Facilitates fellow's career development.
Field supervisor	Technical staff of PHFP secretariat	• Guides fellow during outbreak investigations and other field projects.
		• Reviews fellow's communication products for clearance.
Field coordinator	Technical lead at PHFP secretariat	• Provides overall coordination with MoH, MakSPH, CDC, WHO.
		• Supervises field supervisors.
		• Guides fellow during outbreak investigations and other field projects.
		• Reviews fellow's communication products and reports for clearance.
CDC resident advisor	CDC senior epidemiologist or medical officer	• Serves as the project officer for the cooperative agreement.
		• Provides guidance on the development of PHFP.
		• Mentors fellow during high-profile or complex field investigations.
		• Assumes overall responsibility for the clearance of fellow's communication products.

### Monitoring and Evaluation of Progress

The fellowship program continuously monitors fellows' progress and performs quarterly and annual evaluation and documentation of the fellows' achievements. The evaluation is jointly conducted by host and academic mentors, technical staff at the PHFP secretariat, and the fellow, based on set criteria using a checklist. In addition, program staff conduct supervisory visits to the host sites on an as-needed basis to support the fellow in accomplishing his or her objectives and for problem solving.

At the end of the 2-year period, fellows who have achieved all the deliverables prepare a portfolio outlining their achievements during the fellowship. At the final defense session, fellows describe their achievements during their 2-year period of training in service and present a completed project that has significantly contributed to disease control and public health improvement. A fellow who successfully passes the defense receives a certificate of completion.

## Results

### Enrollment

The program was launched in January 2015 with 10 fellows in the first cohort. As of the end of 2017, 3 cohorts of 31 fellows (10 each in cohorts 2015 and 2016 and 11 in cohort 2017) had been recruited and trained; the fourth cohort of 11 fellows (cohort 2018) has been recruited and will enter the fellowship in January 2018. The enrolled fellows hold various advanced degrees in general public health (MPH or MScPH, 14 fellows); medicine (MMed, 1 fellow); epidemiology or clinical epidemiology (MSc, 4 fellows); entomology, parasitology, or zoology (MSc, 2 fellows); international health or public health (MSc, 2 fellows); health services research and management (MSc, 2 fellows); and other fields (1 fellow each): veterinary preventive medicine (BVM), medical microbiology (MSc), infectious disease management (MSc), and health policy (MSc). Applications increased from 44 in 2015 to 195 in 2018. Fellows have so far been placed in 17 ministry of health priority areas (Table 3). The first cohort fellows have graduated, and second cohort fellows will be graduating in January 2018.

**Table 3.** Placement Sites for Fellows of the Uganda Public Health Fellowship Program, Cohorts 2015 to 2017

*Placement Site*	*Number of Fellows*
**Ministry of Health**	
National Malaria Control Program	3
Division of Health Information	3
Uganda National Expanded Program on Immunization	3
Epidemiology and Surveillance Division	2
STD/AIDS Control Program	2
Mental Health and Substance Abuse	2
National Health Laboratory Services	2
National Tuberculosis and Leprosy Program	2
Reproductive Health Division	2
Neglected Tropical Diseases Program	2
Emergency Operations Centre/Antimicrobial Resistance	1
**Ministry of Health–Affiliated Institutions**	
Uganda Cancer Institute	2
Mildmay Uganda	1
Uganda Virus Research Institute	1
**District/City Health Office**	
Tororo District Health Office/BASIIN Study	1
Rakai District Health Office/Rakai Health Sciences Program	1
Kampala Capital City Authority	1
**Total placements in the 17 priority areas in MoH**	31

### Field Projects

**Figure 1.**
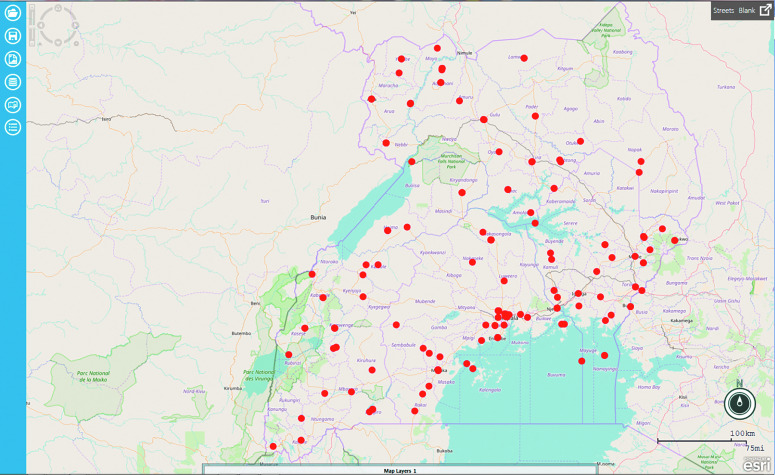
Countrywide Distribution of Projects

Over the past 3 years, the 31 PHFP fellows have completed 153 applied epidemiology projects, of which 60 were outbreak investigations and 12 were emergency refugee health assessments. On average, each fellow participated in 10 field projects, including 6.6 outbreak investigations and 0.51 emergency public health assessments (Table 4). These field investigations have been conducted throughout the country and involved high-priority diseases such as viral hemorrhagic fevers, malaria, tuberculosis, and HIV/AIDS, and diseases targeted for elimination or eradication (eg, Guinea worm disease, human African trypanosomiasis, and measles) and pathogens of bioterrorism potential (such as cholera and anthrax) (Figure 1).

**Table 4.** Achievements by the Fellows of the Uganda Public Health Fellowship Program, 2015-2017

	*Mean Number of Projects and Products, by Cohort*			
	*2015*	*2016* ^a^	*2017* ^b^	*All 3 cohorts* ^c^
**Field projects participated in**	**9.2**	**9.6**	**5.8**	**10**
Outbreak investigations	5.5	5.7	4.3	6.6
Emergency public health assessments	0.30	0.50	0.36	0.51
Public health surveillance projects	1.5	1.4	1.0	1.6
Applied epidemiologic studies	1.8	1.2	0.091	1.1
Cost analysis of outbreaks^d^	0.10	0.10	0.091	0.13
Quality improvement projects^d^	0	0.70	0	0.23
**Scientific publications co-authored**	**9.2**	**6.5**	**7.4**	**10**
Peer-reviewed, published, or accepted	3.4	0.20	0	1.2
Peer-reviewed, submitted, not published yet	1.7	2.6	3.5	3.9
MoH Quarterly *Epi Bulletin* articles^e^	4.1	3.7	3.8	5.2
**Presentations made at scientific conferences**	**5.8**	**4.9**	**2.3**	**5.1**
International conferences	2.3	2.1	0.18	1.6
National conferences	3.5	2.8	2.1	3.5
**Awards received**	**4** ^f^			

a One of the 10 fellows dropped out; calculation of means was based on 10 fellows.

b Cohort 2017 fellows have been in training for only 1 year as of this writing.

c In calculating the means for all 3 cohorts, the numbers for cohort 2017 fellows are multiplied by 2 because they have finished 1 year of the 2-year fellowship.

d Cost analysis and the quality improvement projects were first introduced in 2016.

e These only included articles published in the *Quarterly Epidemiological Bulletin* by MoH and did not include articles published in the *Weekly Surveillance Bulletin* and program-specific bulletins.

f One of the awards, the CDC Director's Award for Excellence in Epidemiology and Public Health Response, was given to the program, not an individual fellow.

### Strengthening Global Health Security

During the past 5 years, the ministry of health has established PHEOC, ESD, and PHFP as the 3 pillars for ensuring that alerts or verified disease outbreaks are promptly reported, responded to, and investigated in real time, and that cost-effective interventions are recommended to control the outbreaks and generate evidence for prevention of future occurrences.

PHFP leads the investigative arm for outbreak response at the ministry of health. Of the 153 projects completed by PHFP over the past 3 years, 78 (51%) involved high-priority diseases, such as potential bioterrorism agents,^19^ epidemic-prone diseases,^20^ and other priority diseases for Uganda (Table 5). Outbreak reports prepared by fellows are routinely submitted to the PHEOC and presented at the National Task Force for Epidemic Preparedness and Response, which helped to guide outbreak prevention and control. The prompt epidemiologic investigations conducted by the PHFP have resulted in shortened time to identify dangerous pathogens and prevented potential spread of outbreaks. For example, due to timely investigation and prompt response, a yellow fever outbreak that occurred in 2016 was confirmed within 12 days and controlled within 3 weeks after the outbreak was reported. In comparison, the previous yellow fever outbreak in Uganda in 2010 took 40 days to confirm and 3 months to control.^11,12^ Because of its stellar achievements and performance, PHFP won the CDC Director's Award for Excellence in Public Health and Response at the 2017 EIS conference.^21^

**Table 5.** Investigations Involving High-Priority Pathogens Conducted by Fellows of Uganda Public Health Fellowship Program, 2015-2017

*Pathogen*	*Number of Projects*
**Potential bioterrorism agents**	**29**
Category A	6
Anthrax	2
Viral hemorrhagic fevers	4
Category B	23
Brucellosis	1
Cholera	10
Food safety threats	12
**Epidemic-prone diseases** ^a^	**20**
Yellow fever	1
Meningococcal disease	2
Influenza (avian & human)	2
African trypanosomiasis	1
HIV/AIDS	14
**Other priority diseases**	**29**
Measles	17
Malaria	6
Tuberculosis	5
Hepatitis B	1
**Total**	**78**

a Cholera, also an epidemic-prone disease, is counted as a potential bioterrorism agent.

In addition to the PHFP, MakSPH has had an MPH program that has been training field epidemiologists since 1994. Also, in 2016, the ministry of health in partnership with CDC established a Frontline FETP to train public health workers at the district level on the detection and initial control of outbreaks.

All of these efforts have positioned the ministry of health itself on the right path of building a pool of field epidemiologists with competencies to strengthen the country's capacity in epidemiology, surveillance, and outbreak response, and to enable the country to appropriately address high-priority public health threats. These efforts are the cornerstone of the successes registered in addressing public health threats that threaten global health security. The ministry of health has achieved this through working in close partnership with other relevant ministries, agencies, and partners with mandates to implement emergency preparedness and response.

### Contribution to HIV Elimination

PHFP fellows have completed multiple projects related to the epidemiology of HIV/AIDS, which provided valuable epidemiologic evidence to guide programming to achieve the “90-90-90 targets.”^6^ These projects examined issues and risk factors related to HIV/AIDS services uptake among young populations, virological nonsuppression, quality of care in HIV service delivery, HIV/AIDS care services for high-risk population groups, knowledge and behavior related to HIV among fishing communities, use of mother-to-child transmission services, quality of reporting for Option B+, and HIV services in the refugee populations.

### Contribution to PMI Targets

Fellows who have been placed in the National Malaria Control Program or with terms of reference addressing malaria control activities have made contributions to achievement of PMI targets and the ministry of health malaria elimination plan.^7^ The achievements include producing the Uganda National Malaria Quarterly Bulletin; applying QGIS to generate malaria-incidence maps; and establishing malaria surveillance thresholds using data from the Health Management Information System. Other malaria-related projects included association between vector control interventions and malaria incidence, malaria morbidity among children under 5 years of age, and malaria vector bionomics study on mosquito behavior and susceptibility to pyrethroids. The analysis of data on malaria incidence supported the decision for providing long-lasting insecticide nets in Uganda.

### Communication of Findings

During 2015 to 2017, PHFP submitted 40 manuscripts for publication in peer-reviewed journals, 17 of which had been published as of December 31, 2017. Findings of some of the published manuscripts have been widely covered by major international media outlets. For example, a manuscript on an investigation of podoconiosis, published in the *American Journal of Tropical Medicine and Hygiene,*^8^ was reported by 29 media outlets as of May 2, 2017, including international media giants such as CNN, the *New York Times,* the BBC, the VOA, *Newsweek*, and NPR.

In addition to the manuscripts, PHFP has had 132 abstracts accepted for presentation at national and international conferences. Notably, every year since 2015, PHFP has had presentations at the international night of the annual EIS conference, the most competitive conference in field epidemiology (including a late-breaker oral presentation in 2015, and both oral and poster presentations in 2016 and 2017). These presentations have won 5 international awards, including the prestigious Jeffrey P. Koplan Award for Excellence in Scientific Poster Presentation (2016).

The program has also supported the ministry of health in reviving and revamping 3 epidemiology bulletins and started 3 new bulletins: *Neglected Tropical Diseases Bulletin, National TB and Leprosy Program Bulletin,* and *Non-communicable Diseases Bulletin*, where the fellows and other ministry of health epidemiologists publish valuable public health information. The quarterly ministry of health *Epidemiological Bulletin* has published 95 articles based on the fellows' projects and the *Weekly Epidemiological Bulletin* has been running since August 2015. Fellows have also written and published many articles in the national newspapers to inform the public about current public health challenges, such as outbreaks and tips on disease prevention.

### Public Health Interventions

Many of the PHFP investigations helped to guide public health authorities to implement interventions. Examples include the multisectoral control effort following the identification and investigation of the typhoid fever outbreak in 2015 in Kampala; the repair of the public water system following investigations of a cholera outbreak in Kasese District in 2015; health worker training and community health education on the prevention of podoconiosis following the investigation in Kamwenge District in 2016; a mass vaccination campaign by the ministry of health after identification of a yellow fever outbreak in south and southwestern Uganda in 2016; multifaceted control efforts after the investigation of a meningitis (serogroup W) outbreak in an institutionalized population in 2016; control efforts implemented by the ministry of health based on epidemiologic evidence generated during the nationwide outbreaks of cholera, malaria, and measles during 2016 and 2017; and the establishment and strengthening of surveillance systems in refugee settlements following assessments and evaluations of surveillance systems in 6 refugee settlements during 2016 and 2017. These interventions have identified and controlled outbreaks at the source in multiple circumstances (eg, the outbreaks of yellow fever in 2016^5^), and prevented the spread of communicable disease to other communities and across borders, thus safeguarding global health security and potentially having contributed to reduction of morbidity and mortality.

### Career Paths of Graduates

One year post-graduation, all 10 graduates of cohort 2015 have been employed in positions where they continue to apply their knowledge and skills in field epidemiology and public health leadership acquired during the fellowship during outbreak responses and other public health activities. Four graduates are currently working in priority programs at the ministry of health, 3 have been retained by PHFP as field supervisors, 1 graduate is working for WHO's NSTOP program in South Sudan, and 2 are working for AFENET as field epidemiologists. All graduates serve as members of the National Rapid Response Team for epidemic preparedness and response. We are also working with the ministry of health to employ more PHFP graduates as permanent field epidemiologists in various priority programs.

## Discussion

The HIV, influenza, Ebola, and Zika epidemics and pandemics during the past half century have taught us repeatedly that, in this increasingly interconnected world, one country's communicable disease problem is every country's problem. Early identification of and prompt response to communicable disease outbreaks are key for their effective and successful containment and control.^8^ Therefore, developing a robust capacity for outbreak detection and control should remain a high priority for the entire global community.

Against this backdrop, the Uganda PHFP was established to face the challenges. As an initiative to develop Uganda's next generation of public health leaders with a special focus on communicable disease control and prevention, PHFP has achieved its initial goals of not only training fellows in practical knowledge and skills but providing valuable services to the ministry of health. During its first 3 years, PHFP has demonstrated the ability to address the gaps identified, as evidenced by the quality products, national and international recognitions, and the adoption of recommendations from their projects. At the same time, PHFP has improved Uganda's capacity to respond to disease outbreaks and other public health emergencies in a timely and effective manner, controlling them at their sources. PHFP has also cultivated core capacities for compliance with the International Health Regulations (IHR) and reinforced the use of evidence for public health practice in priority areas. In so doing, PHFP has served to strengthen the GHSA.

PHFP has also effectively changed the culture of how public health emergencies are responded to in a relatively short period. Prior to the establishment of PHFP, systematic epidemiologic investigations were usually conducted only when outbreaks involved high-priority pathogens such as viral hemorrhagic fevers. For “common” outbreaks such as cholera and measles, general control measures (eg, WASH, vaccination) had been used for outbreak control without an investigation. PHFP has demonstrated that even these common outbreaks need to be investigated, because each outbreak is unique^22^ and the epidemiologic evidence generated during outbreak investigations can be valuable for more targeted and effective interventions and can help prevent future outbreaks and influence public health policies on disease prevention and control. Consequently, the ministry of health now routinely requests the PHFP to conduct investigations when an outbreak occurs, whether it involves a high-priority pathogen or not.

PHFP is among the most productive FETPs around the world, especially in surveillance and outbreak investigations. Such productivity could be attributed to strategically posting fellows to different ministry of health priority areas, the sense of ownership of PHFP by the ministry of health, the close collaboration between PHFP and EOC, and the fellows being part of the National Rapid Response Team. According to an evaluation conducted by CDC in 2014 among field epidemiology training programs, countries that demonstrated the highest ownership of the program provided a wide range of this type of support and relied on the FETP for important public health work. Additionally, the relatively small per-cohort intake that enabled maximum interaction between the fellows and their mentors, and the full-time fellowship approach that enabled fellows to concentrate on their deliverables, have also contributed to PHFP's visible productivity.^23^

Compared to the MPH degree–granting model, in which the students spend a large proportion of their time in didactic training and dissertation work, PHFP's fellowship model has demonstrated a high level of productivity in addressing public health needs of the country. PHFP fellows spend more time working on projects and less time in didactic courses, because they already have a master's degree when they enter the program. They focus more on addressing actual public health needs in the country. For example, a typical MPH student works on 2 to 4 projects during the 2 years of training, whereas a PHFP fellow worked on more than 9 projects on average.

On the other hand, because of the heavy field component, the PHFP costs more than MPH degree–granting programs. However, the return on investment is worthwhile. PHFP provides immediate return on investment through early detection, investigation and control of outbreaks, improvement in surveillance system, and provision of urgently needed data for public health programs. The recent Ebola outbreak in West Africa cost the global community $3.6 billion to respond to and contain and an additional $2.2 billion in GDP loss to Guinea, Liberia, and Sierra Leone.^24^ Had the world community spent a fraction of that sum of money to build a robust system in West Africa for early identification, investigation, and control of outbreaks, the massive economic toll of the Ebola epidemic could have been averted. With accreditation of the PHFP in the near future, and the increasing publicity, the program should be able to attract even more highly capable applicants in the future, as demonstrated by similar fellowship programs such as the EIS at the US CDC and EPIET at ECDC.

The fast absorption of the graduates into leadership positions, including the employment of 4 by ministry of health priority programs, 3 by AFENET and WHO, and the retention of 3 in PHFP, will help with the sustainability of PHFP. The alumni at the program secretariat will offer the best training and learning opportunities for the current fellows. Graduates absorbed into ministry of health priority departments will improve mentorship for incoming fellows assigned to these departments. The prompt public health actions taken based on investigation findings will also help to increase the chances of program sustainability.

Despite the success, PHFP still faces a number of challenges. The program has still not been as well recognized as it should have been. To address this issue, PHFP has worked with the ministry of health to create a new “field epidemiologist” job classification within ministry of health departments, and to have field epidemiology training as a key qualification for certain jobs in the ministry of health. This could act as a model for Africa CDC to advocate for its adoption as established positions in ministries of health in other countries. PHFP is currently also working to obtain formal recognition as a fellowship under the Uganda College of Medicine. An advanced-stage discussion on this matter is going on with the Uganda Medical and Dental Practitioner's Council and the Uganda National Council for Higher Education and other relevant stakeholders. The program is also working to increase its visibility to more stakeholders both within and outside the ministry of health through a multifaceted approach. In addition, PHFP has been preparing to get accreditation by the Training Programs in Epidemiology and Public Health Interventions Network (TEPHINET).

Using evidence generated by PHFP for public health actions is also a challenge, partly due to lack of resources to implement the recommended interventions, especially with regard to measures to prevent outbreaks. PHFP fellows have conducted multiple analyses of the costs associated with outbreaks, which demonstrated time and again that prevention is far more economical than response when it comes to outbreaks. These data will be assembled and shared with government decision makers, international agencies, nongovernmental organizations, and other partners in the hope that more investments will be made in outbreak prevention.

PHFP is currently a project funded by CDC and needs to be fully integrated and institutionalized within the Uganda ministry of health system. Recognizing this need, the ministry of health has made PHFP a key component of the proposed Uganda National Institute of Public Health (UNIPH) by designating it as one of its directorates in its structure. Once the UNIPH is formally established by an act of parliament, it will become an integrated disease control environment in the country, with diversified funding sources from the government, philanthropists, and the private sector, as well as grants and cooperative agreements. The vision is for PHFP to be its capacity-building component, which will provide a competent workforce of field epidemiologists and other health professionals to meet the public health needs of the country. Moreover, PHFP alumni have formed an association called Field Epidemiologists without Borders, which will work closely with UNIPH to ensure the program's sustainability.

## Conclusion

Within the first 3 years of operation, PHFP has established itself as a robust and productive advanced FETP, which has built human resource capacity in public health in Uganda and strengthened global health security through improvement in public health emergency response and generation of evidence to inform decision making by the ministry of health.
